# Supplementary material for: CD301b+ dendritic cell-derived IL-2 dictates CD4+ T helper cell differentiation
Source: Nat Commun. 2025 Feb 26;16:2002. doi: 10.1038/s41467-025-55916-9 (PMC11865452; doi:10.1038/s41467-025-55916-9)
Supplement: Supplementary file 1 — Supplementary Information [file 41467_2025_55916_MOESM1_ESM.pdf]

CD301b$^+$ dendritic cell-derived IL-2 dictates CD4$^+$ T helper cell differentiation

Naoya Tatsumi[1,2], Jihad El-Fenej[1,2], Alejandro Davila-Pagan[1,2], Yosuke Kumamoto[1,2,*]

[1]Center for Immunity and Inflammation, Rutgers New Jersey Medical School, Newark, NJ, USA.
[2]Department of Pathology, Immunology and Laboratory Medicine, Rutgers New Jersey Medical School, Newark, NJ, USA.

*Correspondence: yosuke.kumamoto@rutgers.edu

Inventory of Supporting Information

- Supplementary Figure 1, related to Figure 1
- Supplementary Figure 2, related to Figure 2
- Supplementary Figure 3, related to Figure 3
- Supplementary Figure 4, related to Figure 4
- Supplementary Figure 5, related to Figure 6

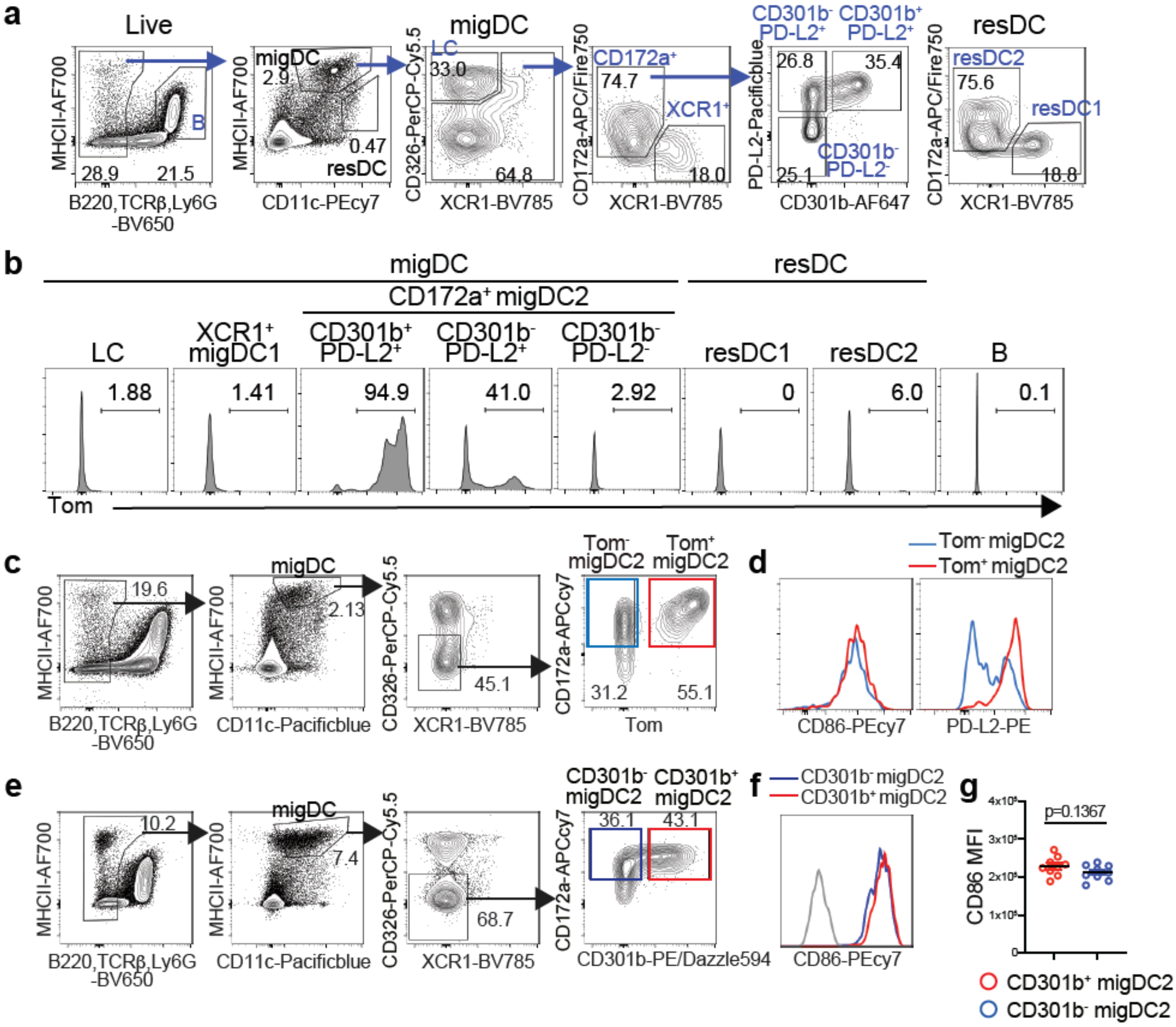

Supplementary Fig. S1. Validation of Mgl2-Cre expression in Mgl2^{cre};R26$^{LSL\text{-}iTom}$ mice.

a. Gating strategies for identifying DC subsets in skin-dLNs. **b**. Representative flow cytometry histograms showing tdTomato (Tom) expression in DC subsets and B cells in skin LNs of naïve Mgl2^{cre};R26$^{LSL\text{-}iTom}$ mice. **c**. Gating strategies for identifying cDC2 cells and Tom expression in Mgl2^{cre};R26$^{LSL\text{-}iTom}$ mice. **d**. Representative flow cytometry histograms of CD86 and PD-L2 expression in cDC2 cells in skin LNs of naïve Mgl2^{cre};R26$^{LSL\text{-}iTom}$ mice. **e**. Gating strategies for identifying cDC2 cells in WT mice. **f**. Representative flow cytometry histograms of CD86 expression in cDC2 cells in skin LNs of naïve WT mice. The gray histogram indicates the binding of isotype control mAb. **g**. CD86 MFI in CD301b^{+} cDC2 and CD301b^{-} cDC2 cells is shown.

Data represent means ± SEM of WT mice (n=9) **(g)** or show representative flow cytometry plots of at least two independent experiments **(a-f)**. Statistical analysis was performed using two-tailed Student's t-test (**g**). Source data are provided as a Source Data file.

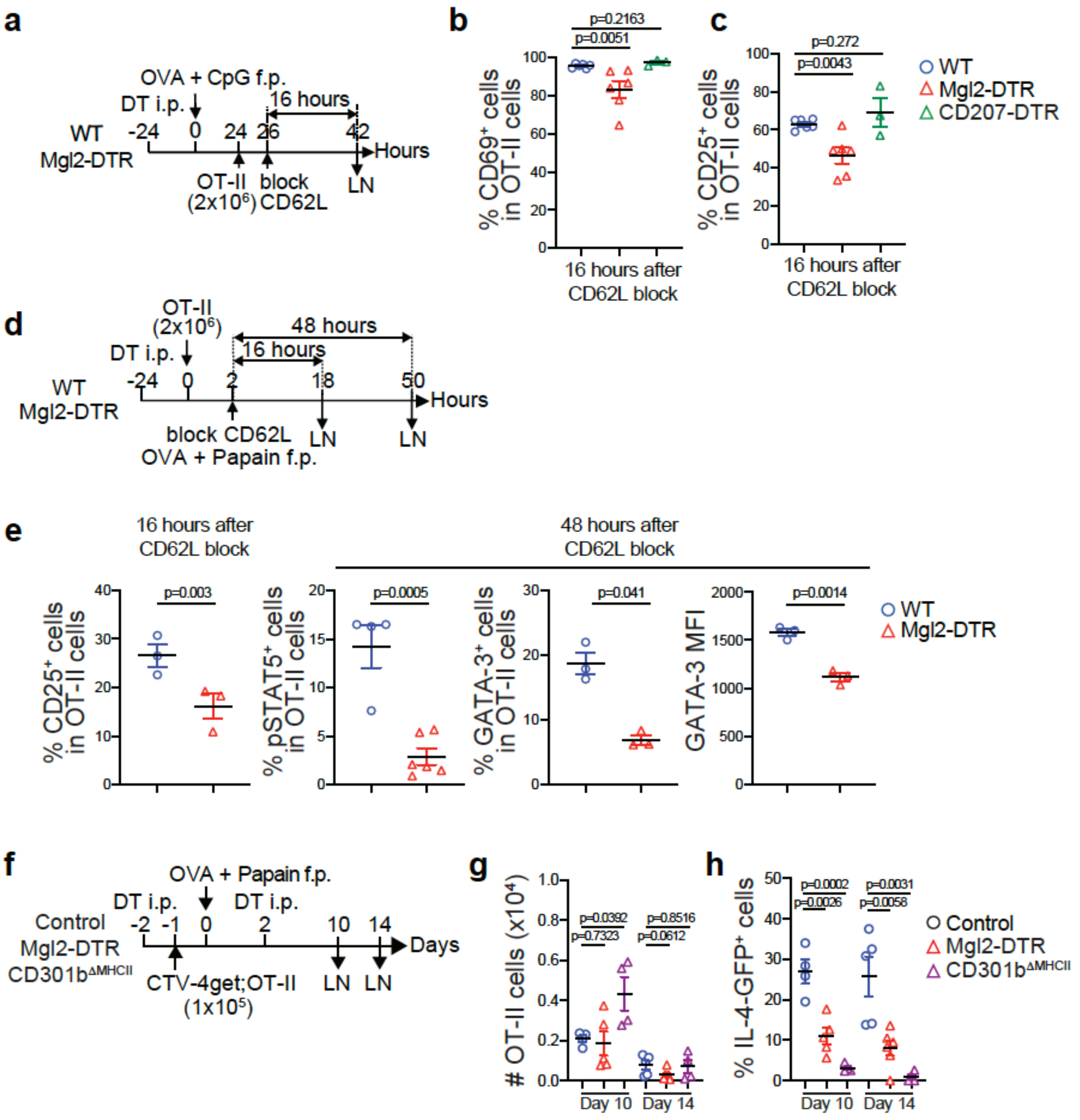

Supplementary Fig. S2. CD301b$^+$ DCs are required for maximal CD25 expression and STAT5 activation in antigen-specific CD4$^+$ T cells.

a. Experimental design. As in **Fig 2a**, but the mice were immunized with OVA plus CpG. The dLNs were harvested 16 hours after the CD62L blockade. **b, c**. Frequencies of CD69$^+$ (**b**) and CD25$^+$ (**c**) cells among the donor OT-II cells are shown. **d**. Experimental design. OT-II cells were transferred into DT-treated Mgl2-DTR or WT mice 24 hours prior to immunization with OVA plus papain in the footpad. Further homing of lymphocytes to LNs was blocked by injecting anti-CD62L mAb retro-orbitally at the time of immunization. The dLNs were harvested at indicated time-points after the CD62L blockade. **e**. Frequencies of CD25$^+$, pSTAT5$^+$, GATA-3$^+$ cells and GATA-3 MFI among the donor OT-II cells at indicated time points are shown. **f**. Experimental design. DT-treated WT and Mgl2-DTR mice were adoptively transferred with 4get;OT-II cells and immunized one day later with OVA plus papain in the footpad. The dLNs were harvested 10 and 14 days after the immunization. Numbers of OT-II cells **(g)** and frequencies of IL-4-GFP$^+$ cells among the donor OT-II cells **(h)** are shown. Data represent means ± SEM of WT (n=6), Mgl2-DTR (n=6), CD207-DTR (n=3) for (**b**), WT (n=3 to 4) and Mgl2-DTR (n=3 to 6) for (**e**), WT (n=4 for day 10 and n=5 for day 14), Mgl2-DTR (n=5 for day 10 and n=6 for day 14), CD301b$^{\Delta MHCII}$ (n=4 for day 10 and n=4 for day 14) for (**g**, **h**). Statistical analyses were performed using two-tailed Student's t-test. Source data are provided as a Source Data file.

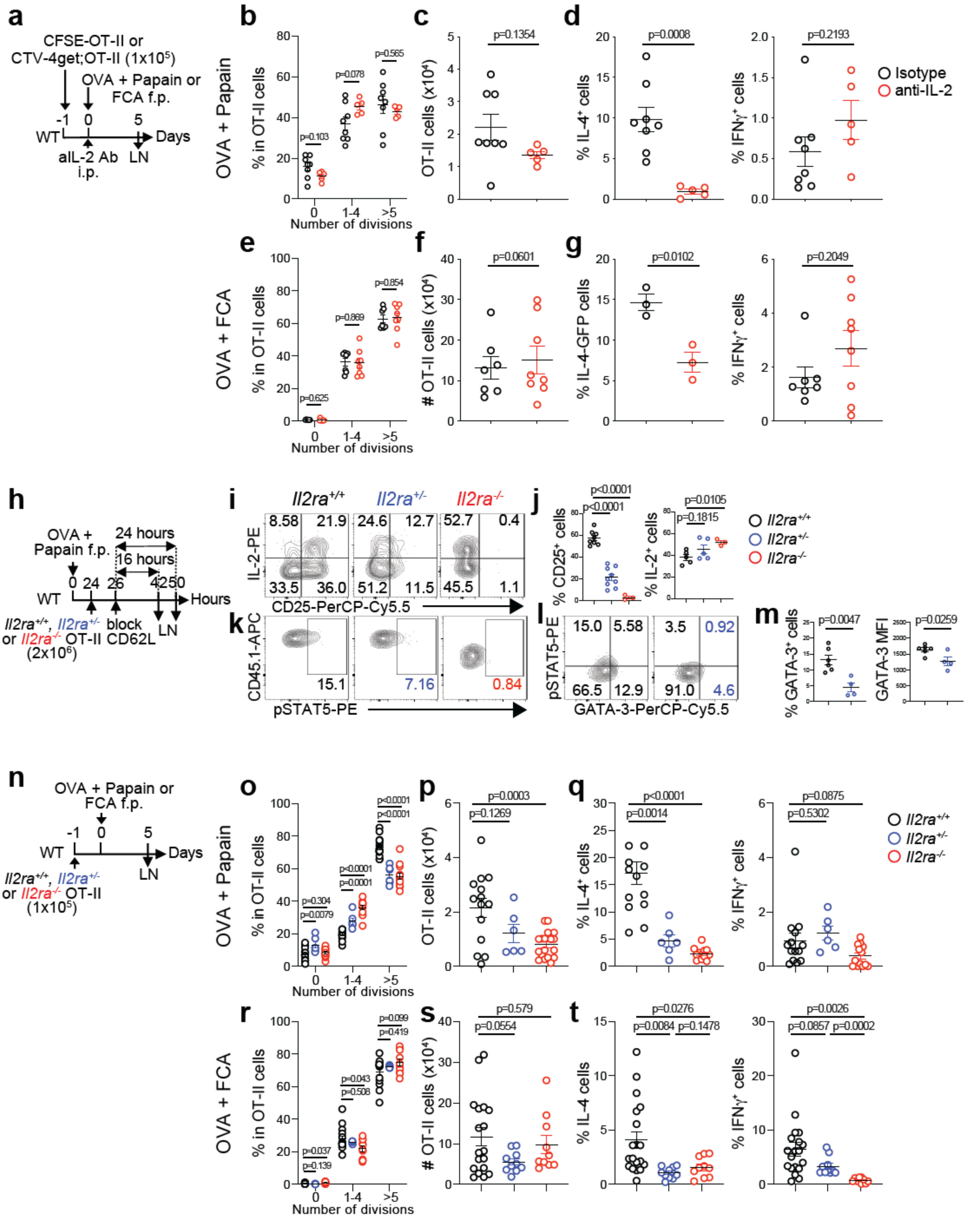
a
CFSE-OT-II or
CTV-4get;OT-II (1x10⁵)
OVA + Papain or
FCA f.p.
WT
Days
aIL-2 Ab
i.p.
LN
b
c
d
OVA + Papain
% in OT-II cells
Number of divisions
OT-II cells (x10⁴)
% IL-4⁺ cells
% IFNγ⁺ cells
Isotype
anti-IL-2
e
f
g
OVA + FCA
OT-II cells (x10⁴)
% IL-4-GFP cells
h
OVA +
Papain f.p.
24 hours
16 hours
Hours
Il2ra+/+, Il2ra+/-
or Il2ra-/- OT-II
(2x10⁶)
block
CD62L
i
j
IL-2-PE
CD25-PerCP-Cy5.5
% CD25⁺ cells
% IL-2⁺ cells
k
CD45.1-APC
pSTAT5-PE
l
GATA-3-PerCP-Cy5.5
m
% GATA-3⁺ cells
GATA-3 MFI
n
OVA + Papain or
FCA f.p.
Il2ra+/+, Il2ra+/-
or Il2ra-/- OT-II
(1x10⁵)
o
p
q
r
s
t
% IL-4 cells

Supplementary Fig. S3. Differentiation of Th1 and Th2 cells in the absence of IL-2R signaling.
a-g. WT mice were transferred with OT-II or 4get;OT-II cells and immunized one day later with OVA plus papain **(b-d)** or OVA plus FCA **(e-g)** in the footpad and injected i.p. with anti-IL-2 neutralizing mAb S4B6-1. The dLNs were harvested 5 days after the immunization and restimulated *ex vivo* with PMA and ionomycin **(a)**. Frequencies of OT-II cells that have undergone indicated number of cell divisions **(b and e)**, numbers of OT-II cells **(c and f)**, and frequencies of IL-4$^+$, IL-4-GFP$^+$, or IFNγ^+ cells among the donor OT-II cells **(d and g)** are shown.
h-m. *Il2ra*$^{+/+}$, *Il2ra*$^{+/-}$, or *Il2ra*$^{-/-}$ OT-II (2×10^6) cells were transferred into WT mice 24 hours after immunization with OVA plus papain in the footpad and allowed to home to LNs for 2 hours, after which further homing was blocked with anti-CD62L mAb **(h)**. The dLNs were harvested 16 **(i-k)** or 24 hours **(l, m)** after the CD62L blockade. For intracellularly staining IL-2, cells were restimulated *ex vivo* with PMA and ionomycin. Representative flow cytometric plots of cell surface CD25 and intracellular IL-2 **(i)**, pSTAT5 **(k, l)**, or GATA-3 **(l)** on the donor OT-II, frequencies of CD25$^+$ and IL-2$^+$ cells **(j)** and GATA-3$^+$ cells and GATA-3 MFI **(m)** among the donor OT-II cells are shown.
n-t. WT mice were transferred with either *Il2ra*$^{+/+}$, *Il2ra*$^{+/-}$, or *Il2ra*$^{-/-}$ OT-II cells separately, and immunized one day later with OVA plus papain **(o-q)** or OVA plus FCA **(r-t)** in the footpad. The dLNs were harvested 5 days after the immunization and restimulated in vitro with PMA and ionomycin **(n)**. Frequencies of OT-II cells that have undergone indicated number of cell divisions **(o and r)**, numbers of OT-II cells **(p and s)**, and frequencies of IL-4$^+$ and IFNγ^+ cells among the donor OT-II cells **(q and t)** are shown.
Data represent means ± SEM of WT treated with isotype (n=8) or anti-IL-2 mAb (n=5) for (**b**-**d**), WT treated with isotype (n=7) or anti-IL-2 mAb (n=8) for (**e**-**g**), WT transferred with Il2ra$^{+/+}$ OT-II (n=8), Il2ra$^{+/-}$ OT-II (n=9) or Il2ra$^{-/-}$ OT-II (n=3) for (% CD25$^+$, **j**), WT transferred with Il2ra$^{+/+}$ OT-II (n=5), Il2ra$^{+/-}$ OT-II (n=4), or Il2ra$^{-/-}$ OT-II (n=3) for (% Il2$^+$, **j**), WT transferred with Il2ra$^{+/+}$ OT-II (n=6), Il2ra$^{+/-}$ OT-II (n=4) for (**m**), WT transferred with Il2ra$^{+/+}$ OT-II (n=14), Il2ra$^{+/-}$ OT-II (n=6), or Il2ra$^{-/-}$ OT-II (n=13) for (**o**-**q**), WT transferred with Il2ra$^{+/+}$ OT-II (n=19), Il2ra$^{+/-}$ OT-II (n=10), or Il2ra$^{-/-}$ OT-II (n=10) for (**r**-**t**), or show representative flow cytometry plots of at least two independent experiments **(i, k, l)**. Statistical analyses were performed using two-tailed Student's t-test **(b-g, j, m, o-t)**. Source data are provided as a Source Data file.

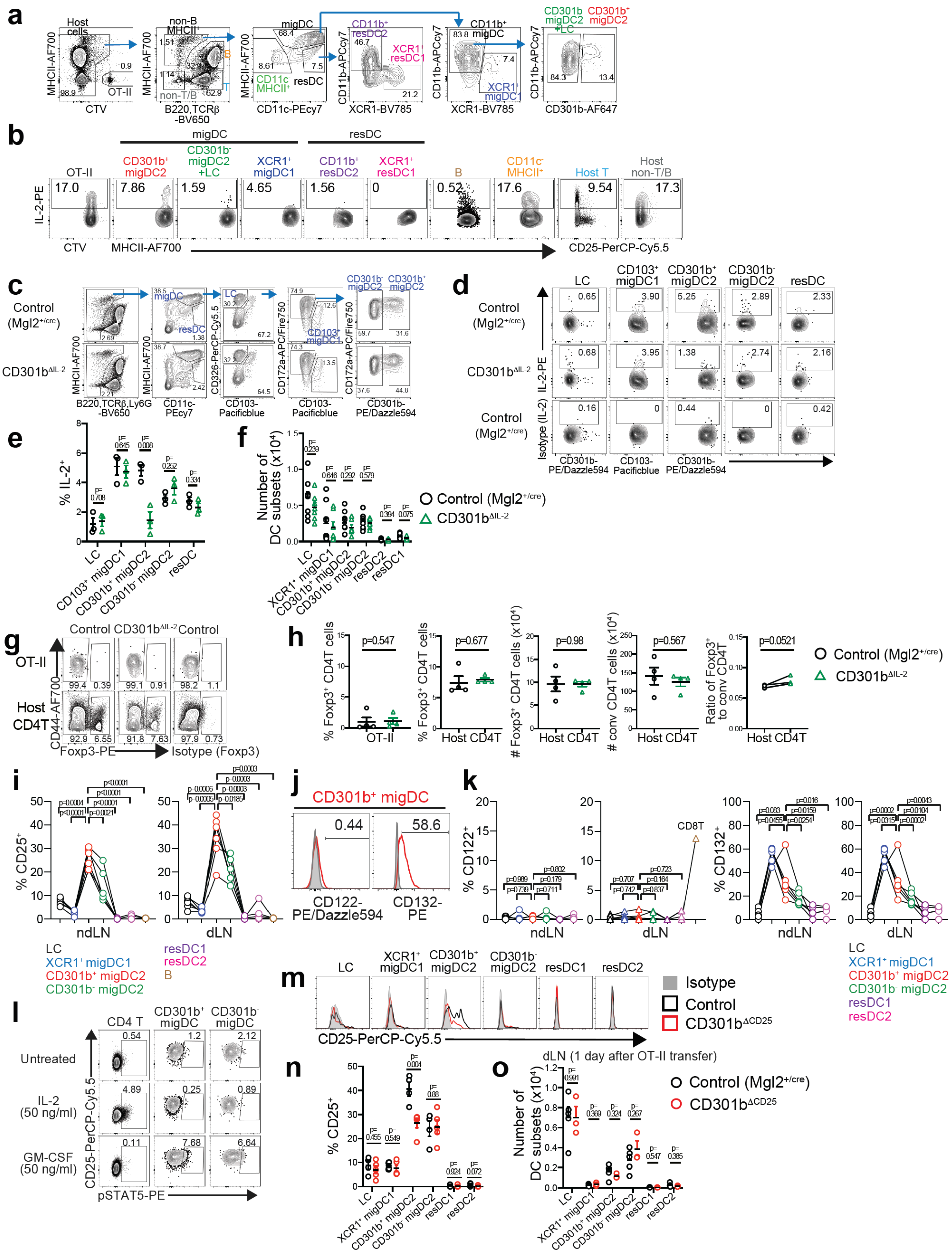

a
b
c
d
e
f
g
h
i
j
k
l
m
n
o
Control (Mgl2+/cre)
CD301bΔIL-2
CD301bΔCD25
CD301b+ migDC
dLN (1 day after OT-II transfer)

Supplementary Fig. S4. Expression of IL-2 and CD25 in CD301b$^+$DCs.
a. Gating strategy for identifying each cell type in **Fig. 4b**.
b. Representative flow cytometry plots of IL-2 expression in each cell type as gated in (**a**).
c-f. Specific deletion of IL-2 in CD301b$^{\Delta IL-2}$ mice. Control (Mgl2$^{+/Cre}$) and CD301b$^{\Delta IL-2}$ mice were immunized with papain in the footpad. The dLNs were harvested 24 hours after the immunization and restimulated ex vivo with PMA and ionomycin for intracellularly staining IL-2. DC subsets were gated as in (**c**). Flow cytometry plots (**d**), frequencies (**e**) of IL-2 in DC subsets, and numbers of each DC subset (**f**) are shown.
g, h. Control (*Mgl2$^{+/Cre}$*) and CD301b$^{\Delta IL-2}$ mice were transferred with 1x10^5 CFSE-labeled OT-II cells and immunized one day later with OVA plus papain in the footpad. The dLNs were harvested 5 days after the immunization. Representative flow cytometry plots of Foxp3 and CD44 expression in donor OT-II and host CD4$^+$ T cells (**g**), frequencies of Foxp3$^+$ cells among donor OT-II and host CD4$^+$ T cells, numbers of Foxp3$^+$ and Foxp3$^-$ conventional host CD4$^+$ T cells, and the ratio of Foxp3$^+$ to Foxp3$^-$ conventional host CD4$^+$ T cells (**h**) are shown.
i-k. Expression of IL-2R subunits in DC subsets. WT mice were immunized with OVA plus papain in the footpad. The popliteal LNs were harvested 24 hours after the immunization for analyzing the expression of CD25, CD122 and CD132 in indicated cell types. Frequencies of CD25$^+$ and CD25 MFI in each DC subsets (**i**), Flow cytometry histograms of CD122 and CD132 in CD301b$^+$ DCs (**j**), frequencies of CD122$^+$ and CD132$^+$ cells in each DC subset (**k**) are shown. Gray histograms in (**i**) indicate the binding of respective isotype control mAb. In (**i**) and (**k**), lines connect values for the same mouse.
l. Skin LNs were harvested from naïve WT mice and were ex vivo stimulated with IL-2 or GM-CSF for 15 min and then immediately fixed. Flow cytometry plots of pSTAT5 and CD25 expression in CD4$^+$ T cells, CD301b$^+$ DCs, and CD301b$^-$ DCs were shown.
m, n. Specific deletion of CD25 in CD301b$^{\Delta CD25}$ mice. CD25 expression was examined in the skin-dLNs of naïve control (Mgl2$^{+/Cre}$) and CD301b$^{\Delta CD25}$ mice. Flow cytometry histograms (**m**) and frequencies (**n**) of CD25$^+$ cells in DC subsets are shown.
o. As in Fig 4a, but control (*Mgl2$^{+/Cre}$*) and CD301b$^{\Delta CD25}$ mice were used and analyzed for DC numbers in the dLNs. Numbers of each DC subset are shown.
Data show representative flow cytometry plots of at least two independent experiments (**a-d, g, l, m**) or represent means ± SEM of control (n=3) and CD301b$^{\Delta IL-2}$ (n=3) for (**e**), control (n=9) and CD301b$^{\Delta IL-2}$ (n=7) for (**f**), control (n=4) and CD301b$^{\Delta IL-2}$ (n=4) for (**h**), WT (n=5) for (**i**, **k**), control (n=4) and CD301b$^{\Delta CD25}$ (n=5) for (**n**), control (n=5) and CD301b$^{\Delta CD25}$ (n=3) for (**o**). Statistical analyses were performed using two-tailed Student's t-test (**e, f, h, n, o**) or two-tailed paired t-test (**i** and **k**). Source data are provided as a Source Data file.

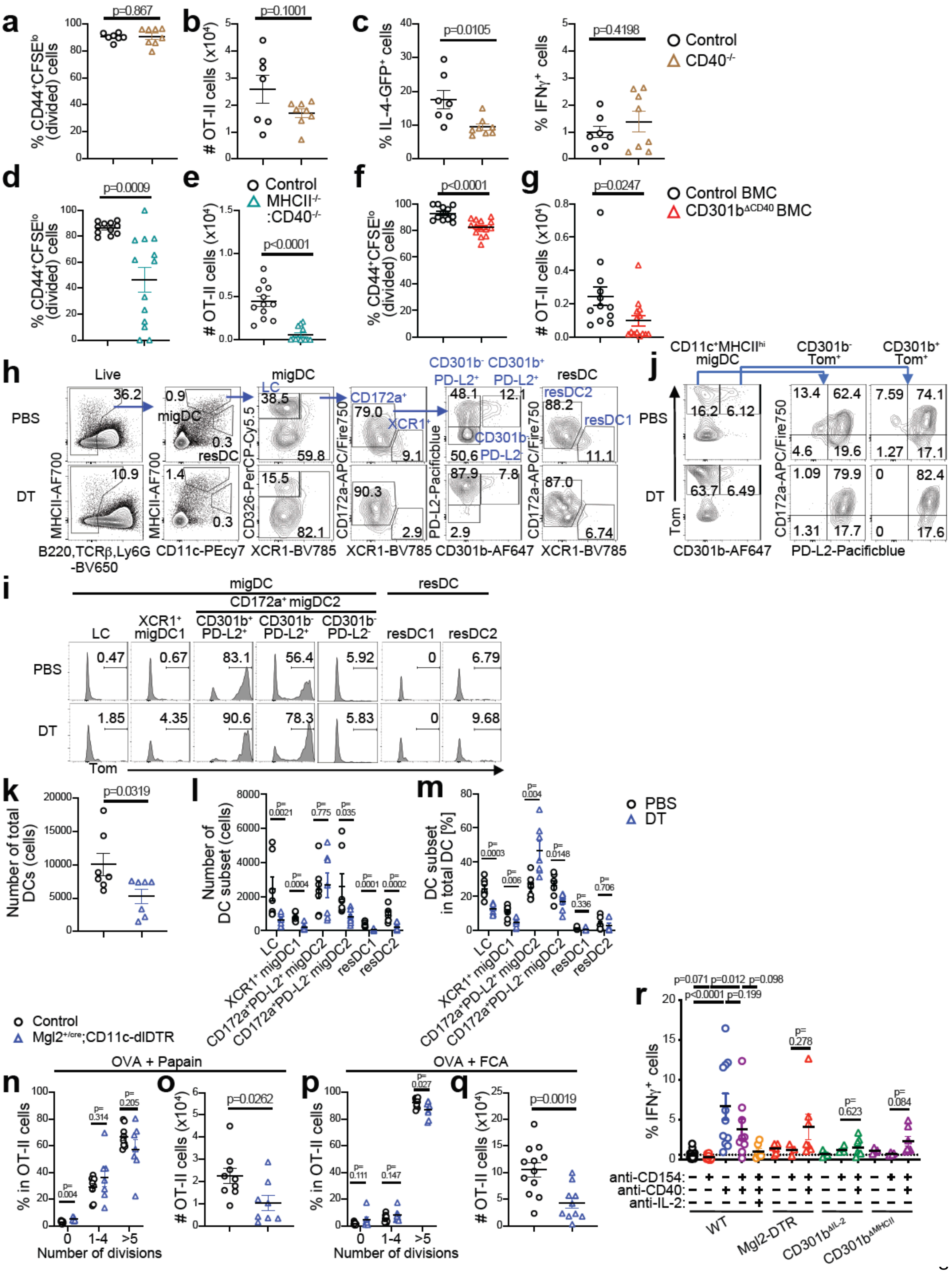

a
% CD44⁺CFSE^lo (divided) cells
p=0.867
b
# OT-II cells (x10⁴)
p=0.1001
c
% IL-4-GFP⁺ cells
p=0.0105
% IFNγ⁺ cells
p=0.4198
Control
CD40-/-
d
% CD44⁺CFSE^lo (divided) cells
p=0.0009
e
Control
MHCII-/-:CD40-/-
OT-II cells (x10⁴)
p<0.0001
f
% CD44⁺CFSE^lo (divided) cells
p<0.0001
g
# OT-II cells (x10⁴)
p=0.0247
Control BMC
CD301b^ΔCD40 BMC
h
Live
migDC
resDC
PBS
DT
LC
CD172a⁺
XCR1⁺
CD301b⁻ PD-L2⁺
CD301b⁺ PD-L2⁺
CD301b⁻ PD-L2⁻
resDC2
resDC1
MHCII-AF700
B220,TCRβ,Ly6G -BV650
CD11c-PEcy7
XCR1-BV785
CD326-PerCP-Cy5.5
CD172a-APC/Fire750
PD-L2-Pacificblue
CD301b-AF647
j
CD11c⁺MHCII^hi migDC
CD301b⁻ Tom⁺
CD301b⁺ Tom⁺
Tom
i
migDC
CD172a⁺ migDC2
resDC
LC
XCR1⁺ migDC1
CD301b⁺ PD-L2⁺
CD301b⁻ PD-L2⁺
CD301b⁻ PD-L2⁻
resDC1
resDC2
k
Number of total DCs (cells)
p=0.0319
l
Number of DC subset (cells)
m
DC subset in total DC [%]
PBS
DT
Control
Mgl2^+/cre;CD11c-dIDTR
OVA + Papain
OVA + FCA
n
% in OT-II cells
Number of divisions
o
# OT-II cells (x10⁴)
p=0.0262
p
% in OT-II cells
Number of divisions
q
# OT-II cells (x10⁴)
p=0.0019
r
% IFNγ⁺ cells
anti-CD154:
anti-CD40:
anti-IL-2:
WT
Mgl2-DTR
CD301b^ΔIL-2
CD301b^ΔMHCII

Supplementary Fig. S5. CD301b$^+$ DC-intrinsic CD40–IL-2 axis is necessary and sufficient for Th2 cell fate instruction.

a-c. WT and CD40$^{-/-}$ mice were transferred with 1x10^5 CTV-labeled 4get;OT-II cells and immunized one day later with OVA plus papain in the footpad. The dLNs were harvested 5 days after the immunization. Frequencies of CD44$^+$ CFSE^{lo} divided cells among the donor OT-II cells **(a)**, numbers of OT-II cells **(b)**, and frequencies of IL-4-GFP$^+$ and IFNγ^+ cells among the donor OT-II cells **(c)** are shown.

d-g. Frequencies of CD44$^+$ CFSE^{lo} divided cells among the donor OT-II cells **(d, f)** and numbers of OT-II cells **(e, g)** in the dLNs of mice shown in **Fig. 6a (d, e)** and **Fig. 6d (f, g)** are shown.

h-m. Mgl2$^{+/cre}$;CD11c-dlDTR;R26$^{LSL\text{-}iTom}$ mice were treated with PBS or DT, and inguinal LNs were harvested 2 days after the DT treatment and stained for DC subsets. Gating strategy for DC subsets including LCs, XCR1$^+$ CD172$^-$ cDC1s, CD301b$^+$ PD-L2$^+$ CD172a$^+$ cDC2s, CD301b$^-$ PD-L2$^+$ CD172a$^+$ cDC2s, CD301b$^-$ PD-L2$^-$ CD172a$^+$ cDC2s, LN-resident XCR1$^+$ cDC1, and LN-resident CD172a$^+$ cDC2 **(h)**, representative flow cytometry histograms of tdTomato expression in each DC subset **(i)**, representative flow cytometry plots of tdTomato and CD301b expression on total DCs (CD11c$^+$MHCII$^+$) and the expression of CD172a and PD-L2 on tdTomato$^+$ CD301b$^+$ and tdTomato$^+$ CD301b$^-$ populations **(j)**, numbers of total DCs **(k)** and each DC subset **(l)**, and frequencies of each subset among total DCs **(m)** in the inguinal LNs 2 days after DT treatment are shown.

n-q. Frequencies of OT-II cells that have undergone indicated number of cell divisions **(n, p)**, numbers of OT-II cells **(o, q)** in the dLNs of mice shown in **Fig. 6i (n, o)** and **Fig. 6j (p, q)** are shown.

r. Frequencies of IFNγ^+ cells among the donor OT-II cells in the dLN of mice shown in **Fig. 6n-p** are shown.

Data represent means ± SEM of control (n=7) and CD40$^{-/-}$ (n=8) for (**a**-**c**), control (n=12) and MHCII$^{-/-}$;CD40$^{-/-}$ mixed BMC (n=12), control (n=12) and CD301b$^{\Delta CD40}$ mixed BMC (n=12) for (**f**, **g**), control (n=7) and Mgl2$^{+/cre}$;CD11c-dlDTR;R26$^{LSL\text{-}iTom}$ (n=7) for (**k**-**m**), control (n=9) and Mgl2$^{+/cre}$;CD11c-dlDTR (n=8) for (**n**, **o**), control (n=12) and Mgl2$^{+/cre}$;CD11c-dlDTR (n=10) for (**p, q**), 3 to 22 mice per each group for (**r**), or show representative flow cytometry plots of at least two independent experiments **(h-j)**. Statistical analyses were performed using two-tailed Student's t-test **(a-g, k-r)**. Source data are provided as a Source Data file.
