# Supplementary material for: CD301b+ dendritic cell-derived IL-2 dictates CD4+ T helper cell differentiation
Source: Nat Commun. 2025 Feb 26;16:2002. doi: 10.1038/s41467-025-55916-9 (PMC11865452; doi:10.1038/s41467-025-55916-9)
Supplement: Supplementary file 2 — Reporting Summary [file 41467_2025_55916_MOESM2_ESM.pdf]

nature portfolio

Corresponding author(s): Yosuke Kumamoto

Last updated by author(s): Dec 6, 2024

# Reporting Summary

Nature Portfolio wishes to improve the reproducibility of the work that we publish. This form provides structure for consistency and transparency in reporting. For further information on Nature Portfolio policies, see our Editorial Policies and the Editorial Policy Checklist.

## Statistics

For all statistical analyses, confirm that the following items are present in the figure legend, table legend, main text, or Methods section.

| n/a | Confirmed | |
|---|---|---|
| ☐ | ☒ | The exact sample size (*n*) for each experimental group/condition, given as a discrete number and unit of measurement |
| ☐ | ☒ | A statement on whether measurements were taken from distinct samples or whether the same sample was measured repeatedly |
| ☐ | ☒ | The statistical test(s) used AND whether they are one- or two-sided<br>*Only common tests should be described solely by name; describe more complex techniques in the Methods section.* |
| ☒ | ☐ | A description of all covariates tested |
| ☒ | ☐ | A description of any assumptions or corrections, such as tests of normality and adjustment for multiple comparisons |
| ☐ | ☒ | A full description of the statistical parameters including central tendency (e.g. means) or other basic estimates (e.g. regression coefficient) AND variation (e.g. standard deviation) or associated estimates of uncertainty (e.g. confidence intervals) |
| ☐ | ☒ | For null hypothesis testing, the test statistic (e.g. *F*, *t*, *r*) with confidence intervals, effect sizes, degrees of freedom and *P* value noted<br>*Give P values as exact values whenever suitable.* |
| ☒ | ☐ | For Bayesian analysis, information on the choice of priors and Markov chain Monte Carlo settings |
| ☒ | ☐ | For hierarchical and complex designs, identification of the appropriate level for tests and full reporting of outcomes |
| ☒ | ☐ | Estimates of effect sizes (e.g. Cohen's *d*, Pearson's *r*), indicating how they were calculated |

*Our web collection on statistics for biologists contains articles on many of the points above.*

## Software and code

Policy information about availability of computer code

| | |
|---|---|
| Data collection | BD LSRII (BD Biosciences) and Attune NxT (Thermo Fisher) for flow cytometric data collection.<br>BD FACS Aria III (BD Biosciences) for cell sorting.<br>NovaSeq 6000 sequencer (Illumina) for DNA sequences data collection. |
| Data analysis | FlowJo (Version 9.3.2 and 10.5.0) for FACS analysis.<br>GraphPad Prism (Version 8) for plotting data points.<br>Cell Ranger, Loupe browser, Partek Flow (Partek Inc), and Ingenuity Pathway Analysis (QIAGEN) for CITEseq data analysis. |

For manuscripts utilizing custom algorithms or software that are central to the research but not yet described in published literature, software must be made available to editors and reviewers. We strongly encourage code deposition in a community repository (e.g. GitHub). See the Nature Portfolio guidelines for submitting code & software for further information.

## Data

Policy information about availability of data

All manuscripts must include a data availability statement. This statement should provide the following information, where applicable:

- Accession codes, unique identifiers, or web links for publicly available datasets
- A description of any restrictions on data availability
- For clinical datasets or third party data, please ensure that the statement adheres to our policy

The CITEseq data used in this study are deposited in GEO database under the accession number GSE281470 (https://www.ncbi.nlm.nih.gov/geo/query/acc.cgi?acc=GSE281470)

## Research involving human participants, their data, or biological material

Policy information about studies with human participants or human data. See also policy information about sex, gender (identity/presentation), and sexual orientation and race, ethnicity and racism.

| | |
|---|---|
| Reporting on sex and gender | There are no human participants or human data in this study |
| Reporting on race, ethnicity, or other socially relevant groupings | N/A |
| Population characteristics | N/A |
| Recruitment | N/A |
| Ethics oversight | N/A |

Note that full information on the approval of the study protocol must also be provided in the manuscript.

# Field-specific reporting

Please select the one below that is the best fit for your research. If you are not sure, read the appropriate sections before making your selection.

☒ Life sciences ☐ Behavioural & social sciences ☐ Ecological, evolutionary & environmental sciences

For a reference copy of the document with all sections, see nature.com/documents/nr-reporting-summary-flat.pdf

# Life sciences study design

All studies must disclose on these points even when the disclosure is negative.

| | |
|---|---|
| Sample size | No statical method was used to determine sample size, but sample sizes were chosen based on the magnitude of the effect being observed, prior experience, and published literatures. |
| Data exclusions | No biological data was excluded from the analysis. Single cell RNA-seq data of low quality was excluded according to pre-established criteria to ensure accurate analyses of the data. |
| Replication | All experiments were repeated at least twice as independent experiments . All results were reproducible. |
| Randomization | Age and sex-matched mice were allocated randomly in the experimental groups. |
| Blinding | The investigators were not blinded during in vivo treatment or data collection, as each mouse has an ear marking for genotype identification that prevents blinding. |

# Reporting for specific materials, systems and methods

We require information from authors about some types of materials, experimental systems and methods used in many studies. Here, indicate whether each material, system or method listed is relevant to your study. If you are not sure if a list item applies to your research, read the appropriate section before selecting a response.

## Materials & experimental systems

| n/a | Involved in the study |
|---|---|
| ☐ | ☒ Antibodies |
| ☒ | ☐ Eukaryotic cell lines |
| ☒ | ☐ Palaeontology and archaeology |
| ☐ | ☒ Animals and other organisms |
| ☒ | ☐ Clinical data |
| ☒ | ☐ Dual use research of concern |
| ☒ | ☐ Plants |

## Methods

| n/a | Involved in the study |
|---|---|
| ☒ | ☐ ChIP-seq |
| ☐ | ☒ Flow cytometry |
| ☒ | ☐ MRI-based neuroimaging |

# Antibodies

Antibodies used

For flow cytometry analysis, the following antibodies were used:
anti-mouse CD4-APC/Fire750 (clone RM4-5, BioLegend, cat# 100568) (dilution 1:300)
anti-mouse CD4-BUV395 (clone GK1.5, BD Biosciences, cat# 563790) (dilution 1:300)
anti-mouse CD8α-PerCP/Cyanine5.5 (clone 53-6.7, BioLegend, cat# 100734) (dilution 1:300)
anti-mouse CD8α-Biotin (clone 53-6.7, BioLegend, cat# 100704) (dilution 1:300)
anti-mouse/human CD45R/B220-Biotin (clone RA3-6B2, BioLegend, cat# 103204) (dilution 1:300)
anti-mouse/human CD45R/B220- PE-Dazzle 594 (clone RA3-6B2, BioLegend, cat# 103258) (dilution 1:300)
anti-mouse TCR-beta- Brilliant Violet 510 (clone H57-597, BioLegend, cat# 109234) (dilution 1:300)
anti-mouse TCR-beta-Biotin (clone H57-597, BioLegend, cat# 109204) (dilution 1:300)
anti-mouse Ly6G-Biotin (clone 1A8, BioLegend, cat# 127604) (dilution 1:300)
anti-mouse CD69-PE (clone H1.2F3, BioLegend, cat# 104507) (dilution 1:300)
anti-mouse CD69-Pacific Blue (clone H1.2F3, BioLegend, cat# 104523) (dilution 1:300)
anti-mouse CD25-PerCP/Cyanine5.5 (clone PC61, BioLegend, cat# 102029) (dilution 1:300)
anti-mouse CD25- PE/Cyanine7 (clone PC61, BioLegend, cat# 102015) (dilution 1:300)
anti-mouse/human CD44-Alexa Fluor 700 (clone IM7, BioLegend, cat# 103026) (dilution 1:100)
anti-mouse CD279-PE-Cy7 (PD-1) (clone RMP1-30, BioLegend, cat# 109110) (dilution 1:300)
anti-mouse I-A/I-E (MHCII)-Alexa Fluor 700 (clone M5/114.15.2, BioLegend, cat# 107622) (dilution 1:300)
anti-mouse I-A/I-E (MHCII)-APC (clone M5/114.15.2, BioLegend, cat# 107613) (dilution 1:300)
anti-mouse/human CD11b-Brilliant Violet 711 (clone M1/70, BioLegend, cat# 101242) (dilution 1:300)
anti-mouse/human CD11b- APC-Fire750 (clone M1/70, BioLegend, cat# 101262) (dilution 1:300)
anti-mouse CD11c-PE-Cyanine7 (clone N418, BioLegend, cat# 117318) (dilution 1:300)
anti-mouse CD11c-PE-Dazzle 594 (clone N418, BioLegend, cat# 117347) (dilution 1:300)
anti-mouse CD326-PerCP-Cy5.5 (clone G8.8, BioLegend, cat# 118220) (dilution 1:300)
anti-mouse CD326-PE (clone G8.8, BioLegend, cat# 118205) (dilution 1:600)
anti-mouse CD301b-Alexa Fluor 647 (clone URA1, BioLegend, cat# 146806) (dilution 1:300)
anti-mouse CD301b-APC (clone URA1, BioLegend, cat# 146814) (dilution 1:300)
anti-mouse CD301b-PE-Dazzle 594 (clone URA1, BioLegend, cat# 146816) (dilution 1:300)
anti-mouse CD172a-APC-Fire750 (clone P84, BioLegend, cat# 144030) (dilution 1:150)
anti-mouse CD172a-PE (clone P84, BioLegend, cat# 144011) (dilution 1:300)
anti-mouse CD273 (PD-L2)-PE (clone TY25, BioLegend, cat# 107205) (dilution 1:300)
anti-mouse CD273 (PD-L2)-Brilliant Violet 421 (clone TY25, BioLegend, cat# 107219) (dilution 1:300)
anti-mouse/rat XCR1-PE (clone ZET, BioLegend, cat# 148203) (dilution 1:300)
anti-mouse/rat XCR1-Brilliant Violet 785 (clone ZET, BioLegend, cat# 148225) (dilution 1:300)
anti-mouse CD103-Pacific Blue (clone 2E7, BioLegend, cat# 121418) (dilution 1:300)
anti-mouse CD40-APC (clone 3/23, BioLegend, cat# 124611) (dilution 1:300)
anti-mouse CD80-FITC (clone 16-10A1, BioLegend, cat# 104705) (dilution 1:300)
anti-mouse CD86-PE-Cyanine7 (clone GL-1, BioLegend, cat# 105013) (dilution 1:300)
anti-mouse CD45.1-APC (clone A20, BioLegend, cat# 110714) (dilution 1:300)
anti-mouse CD45.1-APC-Fire750 (clone A20, BioLegend, cat# 110752) (dilution 1:150)
anti-mouse CD45.2-PE (clone 104, BioLegend, cat# 109807) (dilution 1:300)
anti-mouse CD45.2-APC-Fire750 (clone 104, BioLegend, cat# 109851) (dilution 1:300)
anti-mouse IFNγ-Pacific Blue (clone XMG1.2, BioLegend, cat# 505818) (dilution 1:300)
anti-mouse IFNγ-PerCP-Cy5.5 (clone XMG1.2, BioLegend, cat# 505821) (dilution 1:300)
anti-mouse IL-4-PE (clone 11B11, BioLegend, cat# 504104) (dilution 1:300)
anti-mouse IL-4-PE-Dazzle 594 (clone 11B11, BioLegend, cat# 504132) (dilution 1:300)
anti-mouse IL-2-PE (clone JES6-5H4, BioLegend, cat# 503807) (dilution 1:300)
anti-mouse Foxp3-PE (clone MF-14, BioLegend, cat# 126404) (dilution 1:300)
anti-mouse/human Bcl-6-PE-Dazzle 594 (clone 7D1, BioLegend, cat#358509) (dilution 1:200)
anti-mouse/human GATA-3- PerCP-Cy5.5 (clone 16E10A23, BioLegend, cat# 653811) (dilution 1:200)
anti-mouse/human pSTAT5 (Tyr694)-PE (clone SRBCZX, eBioscience, cat# 12-9010-41) (dilution 1:200)
anti-mouse CXCR5-Biotin (clone 2G8, BD Biosciences, cat# 551960) (dilution 1:50)
anti-mouse PSGL-1-PE (clone 2PH1, BD Biosciences, cat# 555306) (dilution 1:300)

For CITEseq analysis, the following DNA-barcoded TotalSeq mAbs were used:
anti-mouse I-A/I-E (MHCII) (clone M5/114.15.2, BioLegend, cat# 107653) (dilution 1:300)
anti-mouse CD11c (clone N418, BioLegend, cat# 117355) (dilution 1:300)
anti-mouse CD301b (clone URA-1, BioLegend, cat# 146817) (dilution 1:300)
anti-mouse CD8α (clone 53-6.7, BioLegend, cat# 100773) (dilution 1:300)

anti-mouse/human CD11b (clone M1/70, BioLegend, cat# 10125) (dilution 1:300)
anti-mouse CD64 (clone X54-5/7.1, BioLegend, cat# 139325) (dilution 1:300)
anti-mouse CD103 (clone 2E7, BioLegend, cat# 121437) (dilution 1:300)
anti-mouse CD172a (clone P84, BioLegend, cat# 144033) (dilution 1:300)
anti-mouse Ly6C (clone HK1.4, BioLegend, cat# 128047) (dilution 1:300)
anti-mouse/rat XCR1 (clone ZET, BioLegend, cat# 148227) (dilution 1:300)

For in vivo treatment, the following mAbs were used:
anti-mouse L-Selectin (CD62L) (clone Mel-14, BioXcell, cat# BE0021)
anti-mouse IL-2 (clone S4B6-1, BioXcell, cat# BE0043-1)
anti-mouse CD154 (clone MR1, BioXcell, cat# BE0017-1)
anti-mouse CD40 (clone FGK4.5, BioXcell, cat# BE0016-2)
Rat IgG2a isotype control (clone 2A3, BioXcell, cat# BE0089)

Validation

All antibodies used in this study were obtained from commercial source and validated by the source vendor. Some antibodies were also validated in this study by comparing to staining with isotype controls.

The antibodies used for staining in this study were validated by flow cytometric analysis on immune cells by vendors, as follows:
anti-mouse CD4 (clone RM4-5, GK1.5)
anti-mouse CD8α (clone 53-6.7)
anti-mouse/human CD45R/B220 (clone RA3-6B2)
anti-mouse TCR-beta (clone H57-597)
anti-mouse/human CD44 (clone IM7)
anti-mouse I-A/I-E (MHCII) (clone M5/114.15.2)
anti-mouse CD11c (clone N418)
anti-mouse/rat XCR1-PE (clone ZET)
anti-mouse CD103 (clone 2E7)
anti-mouse CD86 (clone GL-1)
anti-mouse CXCR5 (clone 2G8)
anti-mouse Foxp3 (clone MF-14)
C57BL/6 splenocytes were stained.

anti-mouse CD40-APC (clone 3/23)
anti-mouse PSGL-1-PE (clone 2PH1)
Balb/c splenocytes were stained.

anti-mouse Ly6G-Biotin (clone 1A8)
anti-mouse/human CD11b (clone M1/70)
anti-mouse CD172a-APC-Fire750 (clone P84)
anti-mouse CD172a-PE (clone P84)
anti-mouse CD64 (clone X54-5/7.1)
anti-mouse Ly6C (clone HK1.4)
C57BL/6 bone marrow cells were stained.

anti-mouse CD301b (clone URA1)
C57BL/6 bone marrow derived DCs were stained.

anti-mouse CD80 (clone 16-10A1)
LPS-stimulated (3 days) Balb/c splenocytes were stained.

anti-mouse CD69 (clone H1.2F3)
anti-mouse IL-2 (clone JES6-5H4)
PMA + ionomycin-stimulated (6 hrs) C57BL/6 splenocytes were stained.

anti-mouse IFNγ (clone XMG1.2)
PMA + ionomycin-stimulated (6 hrs) Balb/c splenocytes were stained.

anti-mouse IL-4 (clone 11B11)
PMA + ionomycin-stimulated (6 hours, in presence of brefeldin A) Th2-polarized C57BL/6 CD4-positive cells were intracellularly stained.

anti-mouse CD25 (clone PC61)
anti-mouse CD279 (PD-1) (clone RMP1-30)
Con A-stimulated (2-3 days) Balb/c splenocytes were stained.

anti-mouse CD326-PE (clone G8.8)
TE-71 (mouse thymic epithelial stromal cell line) cells were stained.

anti-mouse CD273 (PD-L2) (clone TY25)
Mouse B7-DC transfected BHK cells were stained.

anti-mouse CD45.1-APC (clone A20)
SJL mouse splenocytes were stained.

anti-mouse CD45.2-PE (clone 104)
BALB/c and SJL splenocytes were stained.

Burkittâ€™s lymphoma cell line, Ramos, was treated with the True-Nuclear™ Transcription Buffer Set, and stained with Bcl-6 (clone 7D1)

anti-mouse GATA-3 (clone 16E10A23)
Human T leukemia cell line Jurkat or Burkitt's lymphoma cell line Ramos were treated with FOXP3 Fix/Perm Buffer Set, and then stained.

anti-mouse/human pSTAT5 (Tyr694) (clone SRBCZX)
IL-2-stimulated (15 minutes) human Th2-polarized CD4+ cells were stained using the Fixation/Methanol protocol.

## Animals and other research organisms

Policy information about studies involving animals; ARRIVE guidelines recommended for reporting animal research, and Sex and Gender in Research

| | |
|---|---|
| Laboratory animals | C57BL/6N (B6), Mgl2-DTR, CD207-DTR, CD11c-dlDTR, CD40-/-, MHCII-/-, Mgl2-Cre, Mgl2-Cre;Rosa26LSL-iTom, Mgl2-Cre;MHCIIfl/fl, Mgl2-Cre;Il2fl/fl, Mgl2-Cre;Il2rafl/fl, Mgl2-Cre;CD11c-dlDTR, Mgl2-Cre;CD11c-dlDTR;Rosa26LSL-iTom, CD45.1;4get;OT-II, CD45.1;Nur77-GFP;OT-II, CD45.1;Nur77-GFP;Rag1;Il2ra;OT-II mice strains on a B6 background were used. For all strains, both male and female mice between 6 to 12 weeks old were used. All mice were maintained at a temperature of 65-75°F (18-23°C) and humidity of 40-60% in a 12-h light/dark cycle with free access to water and food in a SPF facility at Rutgers New Jersey Medical School. |
| Wild animals | This study did not involve wild animals. |
| Reporting on sex | Sex differences were not considered as a biological variable. |
| Field-collected samples | This study did not involve samples collected from the field. |
| Ethics oversight | All procedures in mice were performed in accordance to protocols approved by the IACUC at Rutgers University. |

Note that full information on the approval of the study protocol must also be provided in the manuscript.

## Flow Cytometry

### Plots

Confirm that:

☒ The axis labels state the marker and fluorochrome used (e.g. CD4-FITC).

☒ The axis scales are clearly visible. Include numbers along axes only for bottom left plot of group (a 'group' is an analysis of identical markers).

☒ All plots are contour plots with outliers or pseudocolor plots.

☒ A numerical value for number of cells or percentage (with statistics) is provided.

### Methodology

| | |
|---|---|
| Sample preparation | LNs were harvested from mice after euthanasia by carbon dioxide and cervical dislocation. LNs were minced and enzymatically digested with 2.5 mg/ml collagenase D (11088882001, Sigma) in complete RPMI-1640 medium with 10% heat-inactivated fetal bovine serum (FBS) at 37°C for 30 min. The cells were washed with PBS containing 2mM EDTA, and then stained for dead cells with cell viability dye (Zombie Aqua or Zombie UV, BioLegend) in PBS on ice for 20 min. The cells were washed with 2mM EDTA/PBS, incubated with 10 µg/mL anti-CD16/CD32 (2.4G2, BioLegend) on ice for 10 min to block non-specific antibody binding, and stained with fluorochrome-conjugated mAbs on ice for 20 min.<br><br>For intracellular cytokine staining, LN single cell suspensions were stimulated in a 96-well round-bottom plate with Cell Stimulation Cocktail containing PMA and ionomycin (eBioscience 00-4970-03, Thermo Fisher or 423302, BioLegend) at 37°C for 1 hour, and then incubated for another 5 hours at 37°C with additional Protein Transport Inhibitor Cocktail containing Brefeldin A and Monensin (eBioscience 00-4980-03, Thermo Fisher). Cells were then fixed and permeabilized with BD Cytofix/Cytoperm Kit (BD Biosciences) and incubated with anti-cytokine mAbs for 30 min on ice.<br><br>For intranuclear Foxp3 staining, cells were fixed and permeabilized with Foxp3 Transcription Factor Fixation/Permeabilization Buffer (eBioscience 00-5521-00) on ice for 30 min and incubated with anti-Foxp3 mAb on ice for 30 min.<br><br>For staining phosphorylated STAT5 and GATA-3, LNs were fixed with BD PhosflowTM Fix Buffer I (BD Biosciences) at 37°C for 10 min immediately after the harvest and permeabilized with BD PhosflowTM Perm Buffer III (BD Biosciences) on ice for 30 min. After blocking with anti-CD16/CD32 (2.4G2, BioLegend), cells were stained for cell surface molecules on ice for 20 min, washed with PBS containing 2 mM EDTA, and then incubated with anti-pSTAT5 and anti-GATA-3 mAbs at room temperature (20°C–25°C) for 16 hours. |
| Instrument | BD LSRII (BD Biosciences)<br>Attune NxT (Thermo Fisher) |

| | |
|---|---|
| | BD Aria III (BD Biosciences) |
| Software | FlowJo (Version 9.3.2 and 10.5.0) |
| Cell population abundance | Live MHCIIhi CD11c+ B220- cells of skin LNs were sorted by BD FACS Aria III for CITEseq analysis. The purity of the sorted population were over 95% purity. |
| Gating strategy | For all experiments, all LN cells were first gated by FSC-A and SSC-A to exclude debris, after which doublets were gated out using FSC-A and FSC-H. Dead cells were excluded by staining with Zombie-Aqua or Zombie-DAPI live/dead staining dye (BioLegend). The live singlet cells were gated as follows:<br>Donor OT-II (CD8-TCRb+CD4+CD45.1+ or B220-CD8-MHCII-CD4+CD45.1+).<br>DC (B220-TCRb-CD11c+MHCII+) exemplified in Fig 4B and Extended Fig 3H.<br>Host CD44+ CD4T cells (B220-CD8-CD45.1-CD44+CD4+).<br>The parental gating for flow cytometric plots in figures were indicated in the text.<br>Appropriate isotype control mAbs were used to define positive and negative staining cells in key experiments, while other gating was performed according to previously published strategies. |

☒ Tick this box to confirm that a figure exemplifying the gating strategy is provided in the Supplementary Information.
